# Protective effect of bee pollen in acute kidney injury, proteinuria, and crystalluria induced by ethylene glycol ingestion in rats

**DOI:** 10.1038/s41598-022-12086-8

**Published:** 2022-05-19

**Authors:** Asmae Elghouizi, Noori Al-Waili, Nawal Elmenyiy, Salma Elfetri, Abderrazak Aboulghazi, Ahmed Al-Waili, Badiaa Lyoussi

**Affiliations:** 1 Laboratory of Natural Substances, Pharmacology, Environment, Modeling, Health, and Quality of Life (SNAMOPEQ), Department of Biology, Faculty of Sciences Dhar Mehraz, Sidi Mohamed Ben Abdellah University, 30000 Fez, Morocco; 2 New York Medical Care for Nephrology, Queens, NY 11418 USA

**Keywords:** Biochemistry, Plant sciences, Nephrology, Urology

## Abstract

Oxidative stress plays a role in hyperoxaluria-induced kidney injury and crystallization. Bee pollen is a hive product with a high content of antioxidants. The antioxidant content and protective effect of bee pollen extract (BPE) against ethylene glycol (EG) induced crystalluria, and acute kidney injury (AKI) were investigated. The effect of BPE on the EG-induced liver injury and proteinuria was also examined. Ten groups of male Wister rats were treated daily with vehicle, cystone, BPE (100, 250, and 500 mg/kg b.wt.), and group 6–9 treated with EG, EG + BPE (100, 250, and 500 mg/kg b.wt.) and group 10 EG + cystone. The dose of EG was 0.75% v/v, and the dose of cystone was 500 mg/kg b.wt. On day 30, blood and urine samples were collected for analysis. Kidneys were removed for histopathological study. The antioxidant activity of BPE was assessed, and its total phenols and flavonoids were determined. EG significantly increased urine parameters (pH, volume, calcium, phosphorus, uric acid, and protein), blood urea, creatinine, and liver enzymes (*P* < 0.05). EG decreased creatinine clearance and urine magnesium and caused crystalluria. Treatment with BPE or cystone mitigates EG's effect; BPE was more potent than cystone (*P* < 0.05). BPE increases urine volume, sodium, and magnesium compared to the control and EG treated groups. BPE reduces proteinuria and prevents AKI, crystalluria, liver injury, and histopathological changes in the kidney tissue caused by EG. BPE might have a protective effect against EG-induced AKI, crystalluria, proteinuria, and stone deposition, most likely by its antioxidant content and activity.

## Introduction

Acute kidney injury (AKI) and proteinuria are common and associated with significant morbidity and mortality in the clinical setting, where there is no effective intervention to manage them. Ethylene glycol (EG) ingestion increases serum and urine oxalate levels that cause AKI, renal inflammation, and tubular cell toxicity^1,2^. We found that propolis, a bee product with high antioxidant activity, prevents proteinuria and AKI induced by EG^3^. Other studies showed that EG causes acute liver injury^3–5^.

Urolithiasis is a common clinical problem associated with mineral deposition in the urinary system**.** It is a multifactorial disorder resulting from epidemiological, biochemical, and genetic risk factors^6^. Therapy includes allopurinol, citrate, cystone, and thiazide diuretics; however, these are not always effective. Hyperoxaluria is the most crucial factor in calcium oxalate stone formation^7,8^. Increased urinary pH, calcium, phosphate, uric acid, and decreased urinary citrate, magnesium, and volume play a significant role in stone formation^9^.

EG is a synthetic chemical liquid used in almost all radiator fluid products and used as solvent, emulsifier, or surfactant**.** Ingestion of EG causes renal injury, and exposure to this solvent in industries causes impairment in liver and kidney functions^10,11^. EG is a metabolic precursor of oxalate, and the oxalate formation starts after 24–72 h of its administration. It causes severe metabolic acidosis and AKI^12^.

Fresh bee pollen represents the pollen produced in the flowers collected by *Apis mellifera* honey bees’ workers from various floral sources. Bees add sugars from nectar and enrich with their substances, which bind the grains together into small pellets and bring them back to the hive on their wings and legs. Bee pollen is a vital hive product gaining attention as a functional food for human use due to its high content of bio-compounds with health-promoting effects, such as minerals, essential amino acids, antioxidants, vitamins, and lipids^13^**.** Bee pollen possesses a wide range of phenolic compounds, such as rutin, quercetin, vanillic acid, and protocatechuic acid, and its composition varies according to its botanical and geographic origins^13,14^. Bee pollen is one of the bee products, including honey, propolis, wax, and royal jelly, mentioned in Holly Books, the Talmud, the Old and New Testaments of the Bible, and the Holy Quran as healers of diseases. In the Surat Al-Nahel (The Bee), it says (Translate the meaning): *And your LORD (Allah) taught the bee to build its cells in the mountains, on the tree, and in men’s habitations; then to eat of all the fruits of the earth, and find with skill the spacious paths of its LORD: there issues from within their bodies a drink of varying colors, wherein is healing for men, verily in this is a sign for those who give thought*.

Cystone is a well-known polyherbal formulation used to treat urinary tract infection and facilitate kidney stone passage; it has antilithic activity in traditional medicine^15–17^. It contains nine different types of plant extract^17^. It protects against experimentally induced urolithiasis in rats^15^. Cystone at a dose of 500 mg/kg caused a significant reversal of sodium oxalate-induced changes in ion excretion and urinary calcium oxalate concentration in rats^18^. It was found that cystone (500 and 750 mg/kg b.wt.) for 28 days has a protective activity against hyperoxaluria-induced oxidative stress and calcium oxalate crystal deposition by improving antioxidant status and increasing urine volume^19^. Furthermore, cystone protects against cisplatin-induced nephrotoxicity^20^. A review of 50 clinical studies showed that cystone improved symptoms, increased urine volume, and reduced the stone-forming constituents in urine without significant side effects^21^.

Hyperoxaluria compromised antioxidant levels and upregulated inflammatory markers^22^. A recently published review discussed the role of polyphenols in urolithiasis management^23^. Bee pollen shows an anti-inflammatory effect^24^. Therefore, the present study investigates the impact of bee pollen extract (BPE) on kidney function and its protective effect against EG-induced AKI, crystalluria, proteinuria, and hepatotoxicity. The result was compared with cystone. Also, the antioxidant content and activity of BPE were studied.

## Results

### Effect of the interventions on renal function and electrolytes

BPE and cystone did not cause significant changes in serum creatinine, phosphorus, calcium, sodium, and potassium in EG untreated groups (Tables 1 and 2). BPE caused a significant elevation of serum protein and a significant lowering of serum magnesium. EG caused a significant elevation of serum creatinine, blood urea, protein, magnesium, phosphorus, sodium, and potassium (*P* < 0.05). Concomitant use of BPE or cystone with EG caused a significant lowering of the parameters compared to EG alone; BPE at higher doses was more potent than cystone.

**Table 1:** Effect of the interventions on blood level of urea, creatinine and protein on day 30.

Groups	Interventions	Creatinine mg/dl	Urea mg/dl	Protein g/dl
EG untreated groups	Control	0.5 ± 0.09	33.5 ± 4.57	4.25 ± 0.38
	Cystone	0.55 ± 0.09	27 ± 3.11	4.45 ± 0.30
	BPE-100 mg	0.5 ± 0.06	23.2 ± 3.9^*^	6.5 ± 0.74^*,#^
	BPE -250 mg	0.55 ± 0.07	25.3 ± 4.35	6.2 ± 0.28^*,#^
	BPE -500 mg	0.55 ± 0.09	32 ± 4.48	6.16 ± 0.53^*,#^
F/P value		0.605/0.66	6.86/0.001	31.2/0.001
EG treated groups	EG	1.17 ± 0.07^*^	47.5 ± 5.23^*^	8.55 ± 0.57^*^
	EG + Cystone	0,7 ± 0.01^β^	39 ± 4.64^β^	7.18 ± 0.40^β^
	EG + BPE 100 mg	0.9 ± 0.11^β,π^	27 ± 4.84^β,π^	7.3 ± 0.43^β^
	EG + BPE 250 mg	0.7 ± 12^β^	26 ± 4.01^β,π^	6.86 ± 0.30^β^
	EG + BPE 500 mg	0.6 ± 0.09^β^	37.5 ± 5.77^β,π^	6.15 ± 0.64^β,π^
F/P value		34.17/0.001	19.34/0.001	19.243/0.001

*P < 0.05 compared to EG untreated-control group.

^#^*P* < 0.05 compared to EG untreated-cystone group.

^β^*P* < 0.05 compared to EG treated group.

^π^*P* < 0.05 compared to EG treated-EG + cystone group.

**Table 2:** Effect of the interventions on blood level of electrolytes on day 30.

Groups	Interventions	Magnesium mg/dl	Phosphorus mg/l	Calcium mg/dl	Sodium mmol/l	Potassium mmol/l
EG untreated groups	Control	2.49 ± 0.35	4.20 ± 0.35	3.22 ± 0.41	149.5 ± 7.11	5.7 ± 1.44
	Cystone	2.68 ± 0.34	4.28 ± 0.31	3.45 ± 0.44	146 ± 3.89	4.98 ± 1.16
	BPE-100 mg	1.79 ± 0.20^*,#^	4.20 ± 0.47	3.46 ± 0.35	147 ± 6.72	4.92 ± 1.3
	BPE -250 mg	1.98 ± 0.23^#^	4.01 ± 0.29	3.48 ± 0.46	148 ± 5.56	5.45 ± 0.84
	BPE -500 mg	1.96 ± 0.25^#^	4.36 ± 0.36	3.91 ± 0.46	145.50 ± 6.27	5.30 ± 1.09
F/P value		10.85/0.001	0.991/0.430	2.23/0.092	0.423/0.790	0.45/0.769
EG treated groups	EG	3.35 ± 0.50^*^	8.55 ± 0.64^*^	8.55 ± 0.43^*^	182.50 ± 7.14^*^	10.75 ± 2.18^*^
	EG + Cystone	3.02 ± 0.45	5.02 ± 0.34^β^	6.92 ± 0.49^β^	152.72 ± 5.33^β^	4.39 ± 0.44^β^
	EG + BPE 100 mg	2.58 ± 0.45	6.55 ± 0.52^β,π^	7.45 ± 0.33^β^	153.50 ± 5.22^β^	4.45 ± 0.62^β^
	EG + BPE 250 mg	2.36 ± 0.23^β^	5.20 ± 0.56^β^	6.97 ± 0.32^β^	150.50 ± 6.09^β^	3.95 ± 0.56^β^
	EG + BPE 500 mg	2.05 ± 0.38^β,π^	4.95 ± 0.44^β^	5.70 ± 0.47^β,π^	152.63 ± 5.22^β^	3.60 ± 0.57^β^
F/P value		10.44/0.001	53.81/0.001	35.52/0.001	41.68/0.001	55.911/0.001

*P < 0.05 compared to EG untreated-control group.

^#^*P* < 0.05 compared to EG untreated-cystone group.

^β^*P* < 0.05 compared to EG treated group.

^π^*P* < 0.05 compared to EG treated-EG + cystone group.

### Effect of the interventions on the liver enzymes

In EG untreated groups, cystone significantly elevated ALP, while BPE significantly decreased AST and ALP (Table 3).EG significantly elevated liver enzymes in EG treated groups (*P* < 0.05). The concomitant use of BPE or cystone with the EG significantly ameliorated EG-induced liver enzymes' elevation; BPE was more potent than cystone *(P* < 0.05).

**Table 3:** Effect of the interventions on liver enzymes on day 30.

Groups	Interventions	AST (U/L)	ALT(U/L)	ALP (U/L)	GGT(U/L)
EG untreated groups	Control	89.94 ± 9.48	9.50 ± 1.91	144 ± 6.63	4.2 ± 1.16
	Cystone	91.64 ± 5.31	10.50 ± 2.23	167 ± 7.43^*^	5.1 ± 0.89
	BPE-100 mg	89.50 ± 4.87	11.29 ± 2.04	145 ± 4.31^#^	4.0 ± 0.69
	BPE -250 mg	68.11 ± 3.35^*,#^	12.57 ± 2.44	134.98 ± 9.88^#^	4.5 ± 1.09
	BPE -500 mg	57.73 ± 6.1^*,#^	12.85 ± 2.35	122.12 ± 4.77^*,#^	4.3 ± 0.63
F/P value		43.99/0.001	2.57/0.066	35.05/0.001	1.535/0.222
EG treated groups	EG	165.50 ± 9.72^*^	34.97 ± 4.35^*^	196 ± 11.88^*^	10.50 ± 1.66^*^
	EG + Cystone	98.33 ± 4.81^β^	18.50 ± 3.08^β^	183.16 ± 4.87	6.45 ± 1.29^β^
	EG + BPE 100 mg	142.2 ± 11.77^β,π^	19.50 ± 3.65^β^	190 ± 9.21	4.50 ± 1.22^β^
	EG + BPE 250 mg	114.5 ± 8.6^β,π^	14.56 ± 1.78^β^	169.90 ± 6.17^β,π^	5 ± 0.89^β^
	EG + BPE 500 mg	93 ± 5.03^β,π^	13.79 ± 3.14^β^	154 ± 9.25^β,π^	4.45 ± 0.88^β^
F/P value		79.23/0.001	41.67/0.001	23.37/0.001	25.99/0.001

*P < 0.05 compared to EG untreated-control group.

^#^*P* < 0.05 compared to EG untreated-cystone group.

^β^*P* < 0.05 compared to EG treated group.

^π^*P* < 0.05 compared to EG treated-EG + cystone group.

### Effect of the interventions on the urine volume and pH

In EG-untreated rats (Table 4), BPE and cytosine caused a significant elevation of urine volume on days 15 and 30 compared to the baseline and the control (*P* < 0.05). BPE caused a significant increase in urinary sodium and chloride and decreased urinary potassium, which means that BPE has a potassium-sparing effect. In the EG-treated groups, EG caused a significant increase in the urine volume, which was significantly potentiated with the concomitant use of the BPE. In the EG-untreated groups, BPE and cystone increased insignificantly *(p* > 0.05) urine pH in EG untreated rats (Table 4). In the EG-treated rats, EG caused a significant elevation of the urine pH, which was mitigated by the concomitant use of either cystone or BPE.

**Table 4:** Effect of interventions on urine volume and pH.

Groups	Interventions	Urine volume (ml)				Urine pH			
		Day 0	Day 15	Day 30	F/P values	Day 0	Day 15	Day 30	F/P values
EG untreated groups	Control	6 ± 1.01	6.69 ± 1.57	7.2 ± 1.52	1.18/0.33	6.55 ± 1.13	6.61 ± 0.72	6.57 ± 0.65	0.02/0.972
	Cystone	5.9 ± 1.5	16.5 ± 3.04^*,α^	22.42 ± 3.03^*,α^	58.07/0.001	6.41 ± 0.91	6.82 ± 0.2	7.16 ± 0.66	1.72/0.21
	BPE-100 mg	5.9 ± 1.04	14.6 ± 2.93^*,α^	19.5 ± 2.25^*,α^	52.15/0.001	6.53 ± 0.95	6.65 ± 0.68	6.89 ± 0.53	0.73/0.49
	BPE -250 mg	6 ± 1.5	15.5 ± 2.13^*,α^	19.5 ± 3.01^*,α^	50.97/0.001	6.45 ± 1.44	6.83 ± 0.66	6.74 ± 0.61	0.288/0.753
	BPE -500 mg	6.4 ± 1.54	15.1 ± 2.9^*,α^	24 ± 3.57^*,α^	63.64/0.001	6.39 ± 0.82	6.57 ± 1	6.83 ± 0.51	0.603/0.55
F/P values		0.12/0.97	14.21/0.001	35.99/0.001		0.03/0.99	0.143/0.96	0.821/0.52	0.12/0.97
EG treated groups	EG	6.5 ± 1.85	15.5 ± 3.8^*,α^	16 ± 2.73^*,α^	19.64/0.001	6.52 ± 0.9	6.99 ± 0.53	7.83 ± 0.46^*,α^	6.35/0.01
	EG + Cystone	5.96 ± 1.8	10.23 ± 2.68^α^	13.2 ± 1.86^b^	16.76/0.001	6.41 ± 1.05	6.86 ± 1.01	6.97 ± 0.39	0.80/0.466
	EG + BPE 100 mg	5.9 ± 1.44	19.5 ± 2.73^π,α^	39.5 ± 4.41^β,π,α^	176.43/0.001	6.61 ± 0.85	6.57 ± 0.63	6.61 ± 0.57	0.005/0.99
	EG + BPE 250 mg	6.32 ± 1.36	18.25 ± 1.12^π,α^	39.5 ± 1.54^β,π,α^	976.14/0.001	6.54 ± 0.86	6.48 ± 0.58	6.53 ± 0.42^β^	0.057/0.94
	EG + BPE 500 mg	6 ± 1.41	22.5 ± 2.34^β,π,α^	42.5 ± 4.29^β,π,α^	229.91/0.001	6.49 ± 0.8	6.56 ± 0.53	6.58 ± 0.84^β^	0.117/0.889
F/P values		0.298/0.87	17.74/0.001	119.1/0.001		0.054/0.99	0.89/0.48	6.14/0.004	0.298/0.87

*P < 0.05 compared to EG untreated-control group.

^#^*P* < 0.05 compared to EG untreated-cystone group.

^β^*P* < 0.05 compared to EG treated group.

^π^*P* < 0.05 compared to EG treated-EG + cystone group.

^α^*P* < 0.05 compared to day 0.

### Effect of the interventions on urine calcium, phosphate, and magnesium

BPE increased urine magnesium in EG-untreated groups compared to the control group (Table 5). Cystone does not affect urine calcium, phosphate, and magnesium. In the EG-treated group, EG caused a significant (*P* < 0.05) lowering of urine magnesium and significant elevation of the urine calcium and phosphate compared to the control group (Table 5). However, treatment with BPE significantly increased urine magnesium and decreased urine calcium and phosphate level when administered with EG compared to the EG-treated group (*P* < 0.05). BPE (500 mg/kg b.wt.) has the highest effect on urine magnesium, calcium, and phosphate levels compared to the cystone treated group and the EG-treated group.

**Table 5:** Effect of the interventions on urine electrolytes on day 30.

Groups	Interventions	Magnesium mg/l	Calcium mg/l	Phosphorus mg/l	Sodium mmol/l	Potassium mmol/l	Chloride mmol/l
EG untreated groups	Control	268.53 ± 10.37	120.19 ± 8.02	53.34 ± 8.53	146 ± 10.48	262.50 ± 16.05	121.50 ± 11.43
	Cystone	281.11 ± 13.96	119.42 ± 9.08	49.72 ± 9.59	243.50 ± 12.32^*^	233.50 ± 13.09^*^	287 ± 12.36^*^
	BPE-100 mg	283.11 ± 12.81	123.38 ± 9.13	48.56 ± 8.48	282.50 ± 9.35^*,#^	209.50 ± 18.32^*^	286 ± 10.03^*^
	BPE -250 mg	286.61 ± 12.02	119.41 ± 9.28	49.18 ± 8.92	389.2 ± 25.36^*,#^	211.45 ± 14.66^*^	309.50 ± 15.98^*^
	BPE -500 mg	290.73 ± 10.58^*^	123.35 ± 10	53.19 ± 6.93	391 ± 29.63^*,#^	225.90 ± 15.76^*^	304.5 ± 19.59^*^
F/P value		3.164/0.03	0.309/0.888	0.563/0.691	180.94/0.001	11.79/0.001	184.03/0.001
EG treated groups	EG	136.91 ± 11.89^*^	165.66 ± 9.51^*^	37.77 ± 8.88^*^	32 ± 9.29^*^	163.91 ± 13.45^*^	82.50 ± 13.76^*^
	EG + cystone	225.15 ± 11.48^β^	124.67 ± 11.16^β^	49.38 ± 9.57	147.22 ± 16.53^β^	223.15 ± 13.95^β^	254.97 ± 17.52^β^
	EG + BPE 100 mg	185.11 ± 12.08^β,π^	155.35 ± 10.12^π^	69.76 ± 8.91^β,π^	132 ± 12.11^β^	159.50 ± 9.81^π^	126.50 ± 17.07^β^
	EG + BPE 250 mg	222.23 ± 11.92^β^	147.29 ± 10.59^β,π^	57.94 ± 11.37^β^	219.5 ± 17.60^β,π^	153 ± 19.15^π^	176 ± 15.01^β^
	EG + BPE 500 mg	234.97 ± 13.77^β^	122 ± 8.86^β^	50.45 ± 9.32	223.5 ± 15.17^β,π^	176.40 ± 13.59^π^	287.39 ± 14.80^β,π^
F/P value		70.00/0.001	20.72/0.001	10.02/0.001	183.50/0.001	23.00/0.001	184.72/0.001

*P < 0.05 as compared to EG untreated-control group.

^#^*P* < 0.05 as compared to EG untreated-cystone group.

^β^*P* < 0.05 as compared to EG treated group.

^π^*P* < 0.05 as compared to EG treated-EG + cystone group.

### Effect of the interventions on the Urinary Excretion of sodium, Potassium, chloride

BPE and cystone significantly increased urine sodium and chloride in EG untreated groups and decreased urine potassium compared to the control group (Table 5). In the EG treated groups, EG significantly decreased urine sodium, potassium, and chloride (*P* < 0.05) (Table 5). However, the concomitant use of BPE or cystone with EG caused a significant increase in the urine sodium, and chloride compared to EG treated group. The effect of BPE was dose-dependent and more potent than cystone.

### Effect of the interventions on urine creatinine, urea, proteins, uric acid, and creatinine clearance

In EG untreated groups, the interventions did not cause significant changes in the urine protein, urea, and uric acid (Table 6) but caused a significant elevation in the creatinine clearance (*P* < 0.05); BPE was more potent than cystone. EG caused a significant elevation in the urine protein, and uric acid and a significant decrease in the urine creatinine and urea and creatinine clearance in the EG treated group (*P* < 0.05). The concomitant use of BPE and cystone with EG decreased urine uric acid and increased urine urea, urine creatinine, and creatinine clearance compared to the EG treated group. Interestingly, BPE or cystone with EG caused a significant lowering in the urine protein, and BPE at a dose of 500 mg was significantly more potent than cystone.

**Table 6:** Effect of the interventions on urine proteins, urea, creatinine, and uric acid and on creatinine clearance on day 30.

Groups	Interventions	Protein mmol/l	Urea mmol/l	Creatinine mmol/l	Uric acid mmol/l	Creatinine clearance (ml/min)
EG untreated groups	Control	22.50 ± 3.84	47.10 ± 4.11	46.20 ± 5.47	7.5 ± 0.9	0.047 ± 0.012
	Cystone	24.50 ± 3.62	45.53 ± 4.83	47 .07 ± 4.8	8.20 ± 1.30	0.138 ± 0.040^*^
	BPE-100 mg	19.78.50 ± 3.12	42.62 ± 5.47	48.98 ± 4.7	8.89 ± 1.35	0.134 ± 0.023^*^
	BPE -250 mg	20.07 ± 3.83	41.90 ± 3.48	49.25 ± 4.31	9.4 ± 1.3	0.124 ± 0.031^*^
	BPE -500 mg	21.78 ± 3.85	42.96 ± 4.45	53.40 ± 4.71	8.32 ± 1.17	0.165 ± 0.039^*^
F/P value		1.681/0.185	1.677/0.1867	1.888/0.143	2.118/0.108	12.424/0.001
EG treated groups	EG	45 ± 3.83^*^	26.94 ± 3.21^*^	19.30 ± 3.86^*^	13.75 ± 1.27^*^	0.017 ± 0.002^*^
	EG + Cystone	32.80 ± 4.17^β^	34.33 ± 3.28	34.80 ± 5.1^β^	10.96 ± 2.14	0.045 ± 0.008^β^
	EG + BPE 100 mg	40.62 ± 4.84^π^	30.39 ± 3.75	24.62 ± 3.41^π^	10.20 ± 2.3	0.084 ± 0.02^β,π^
	EG + BPE 250 mg	34.62 ± 4.64^β^	31.09 ± 3.55	34.62 ± 4.78^β^	11.60 ± 1.83	0.148 ± 0.041^β,π^
	EG + BPE 500 mg	24.12 ± 3.89^β,π^	36.87 ± 4.75^β^	40.12 ± 3.96^β^	9.70 ± 1.47^β^	0.200 ± 0.037^β,π^
F/P value		21.35/0.001	5.499/0.002	13.58/0.001	3.124/0.032	47.89/0.001

*P < 0.05 as compared to EG untreated-control group.

^#^*P* < 0.05 as compared to EG untreated-cystone group.

^β^*P* < 0.05 as compared to EG treated group.

^π^*P* < 0.05 as compared to EG treated-EG + cystone group.

### Urine microscopy and kidney histopathological study

**Figure 1:**
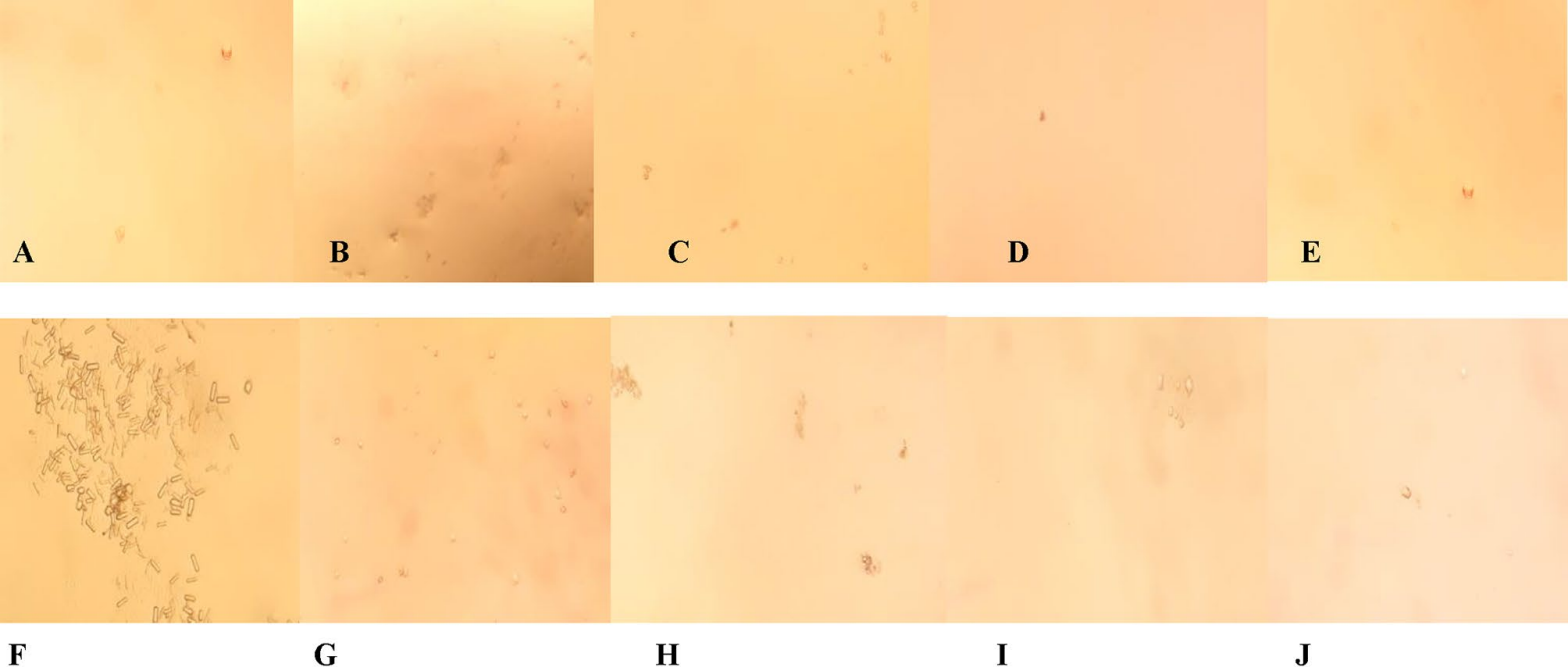
Light microscopic examination of the urine samples under the microscope at 10 × 10X magnification showing multiple crystals in the EG treated group. (**A**) Control group; (**B**) Cystone treated group; (**C**) Bee pollen treated group 100 mg; (**D**) Bee pollen treated group 250 mg; (**E**) Bee pollen treated group 500 mg; (**F**) EG treated group; (**G**) EG + Cystone treated group; (**H**) EG + Bee pollen treated group 100 mg; (**I**) EG + Bee pollen treated group 250 mg; (**J**) EG + Bee pollen treated group 500 mg.

EG causes crystalluria compared to the control groups (Fig. 1). However, the concomitant use of BPE or cystone with the EG markedly decreased crystalluria and was resolved using BPE at a dose of 500 mg/k b.wt./day.

**Figure 2:**
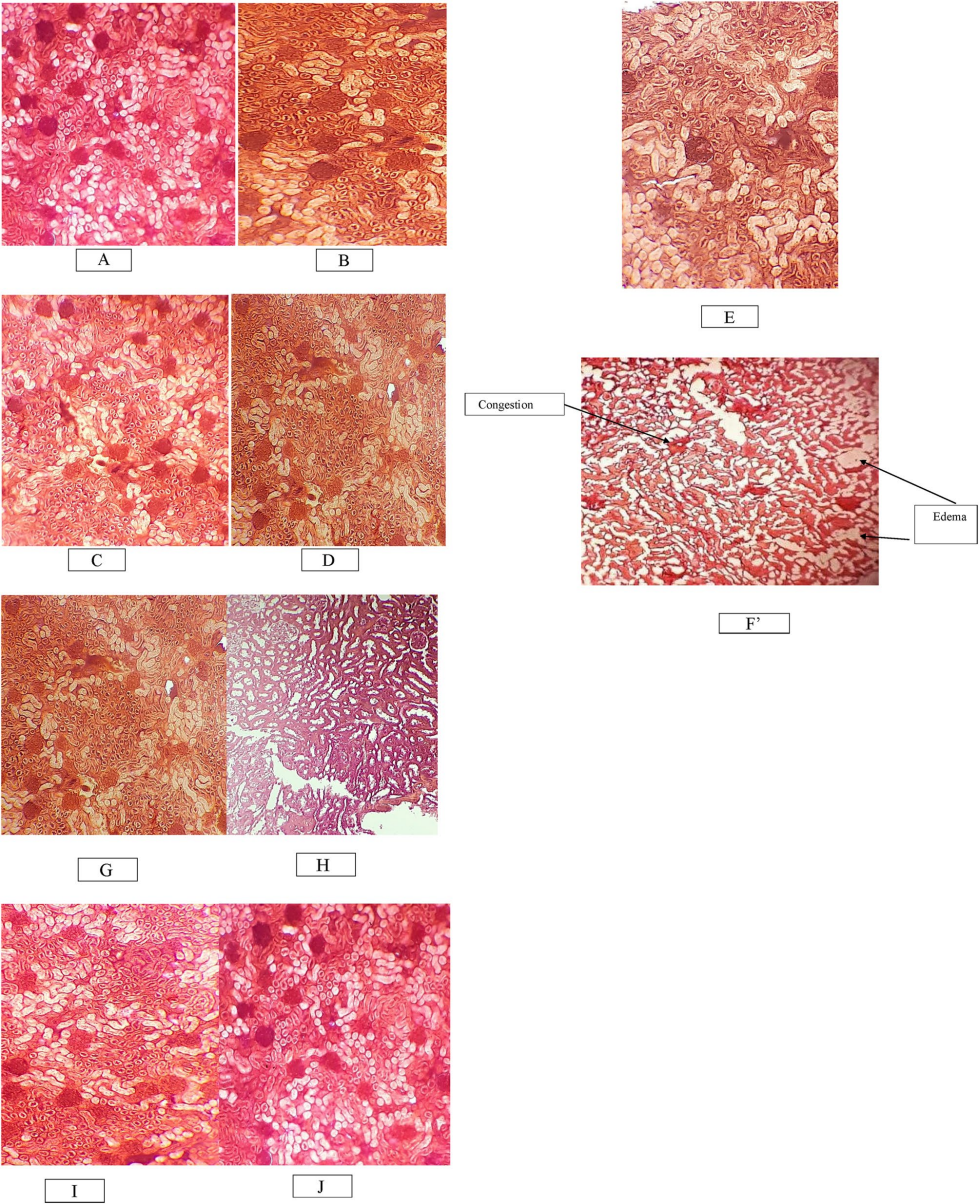
Histopathological examination of kidney slides of different treatment groups seen under a microscope at 10 × 10X magnification. (**A**) Control group; (**B**) Cystone treated group; (**C**) Bee pollen treated group 100 mg; (**D**) Bee pollen treated group 250 mg; (**E**) Bee pollen treated group 500 mg; (**F**) EG treated group; (**G**) EG + Cystone treated group; (**H**): EG + Bee pollen treated group 100 mg; (**I**) EG + Bee pollen treated group 250 mg; (**J**) EG + Bee pollen treated 500 mg.

Regarding the kidney histopathology study, the results showed that EG caused crystal deposits in the tubules, dilatation of the collecting tubules, necrosis of the tubular epithelium, inflammation, edema, and congestion of interstitium (Fig. 2). Interestingly, BPE and cystone treatment markedly prevented these histological changes observed in the EG-treated groups.

### Antioxidant activity assays and Phenolic constituents

BPE showed an important antioxidant capacity towards the DPPH, ABTS and ferric reducing power (0.72 ± 0.031 mg/ml, 0.16 ± 0.04 mg/ml and 0.12 ± 0.087 mg/ml respectively) but these values were significantly (*P* < *0.05*) lower than BHT (0.021 ± 0.01 mg/ml), the Gallic acid (0.019 ± 0.001 mg/ml) and the ascorbic acid (0.03 ± 0.07 mg/ml). The amount of phenols in BPE was 51.35 ± 2.61 mg GAE/gram, flavonoids content was 1.87 ± 0.06 mg QE/gram and total antioxidant capacity was 234.53 ± 2.63 mg AAE /gram.

## Discussion

The effects of EG demonstrated in the present study are similar to its reported impacts elsewhere^3,25^. EG increases urine calcium, phosphate, uric acid, proteins, and oxalate in rats^26^. Another study showed that EG causes renal damage by increasing creatinine, uric acid, and proteins^27^.

Treatment with BPE or cystone increases urine volume. The diuretic activity of BPE might be due to the significant increase in urine sodium and chloride excretion. BPE decreased potassium excretion compared to the control group. Therefore, BPE has a considerable diuretic activity with a potassium-sparing effect. The increase in the urine excretion decreases urine solute concentration and ultimately reduces saturation and stone formation. Thiazide diuretics, part of stone management, reduce urinary supersaturation because of their diuretic and hypocalciuric action^28^. We have found that honey and propolis contain polyphenols and cause a significant diuresis^29^. Studies showed that flavonoids possess diuretic effects^30,31^. This effect might help to explain the diuretic activity of BPE. EG also causes an increase in urine volume. BPE with EG causes a further increment in the urine volume.

BPE causes increased serum protein in EG untreated group, most likely due to its nutritional content. EG caused increased serum protein, which might be due to inflammatory reaction and tissue damage caused by EG. However, BPE mitigates hyperproteinemia caused by EG, which is likely by its antioxidant and anti-inflammatory activity. Further studies are needed to explore the mechanism of action.

The present study showed that BPE significantly decreases urine calcium and phosphate levels, which presumably helps prevent stone formation. An increase in the urine phosphorus and calcium excretion was observed in the EG-treated rats, which led to calcium phosphate crystals. The oxalate presence provides a suitable environment for stone formation where the calcium phosphate induces calcium oxalate deposition^32,33^. Therefore, BPE might reduce stone formation by increasing urine volume and decreasing urine calcium, phosphate, and oxalate crystals, which was evident by the direct microscopic examination of the urine. It was found that magnesium is a well-known inhibitor of calcium oxalate crystallization, and it can reduce the precipitation potential^34,35^. In the present experiment, EG decreases urine magnesium that was increased by BPE or cystone. In earlier observation, we found that propolis, another bee product, also alleviated EG-induced urinary depletion of magnesium^3^.

The administration of EG significantly decreases urine volume, resulting from renal impairment. It was found that EG causes AKI due to proximal tubular cell necrosis that results from several metabolites (glycolaldehyde, glyoxylate, glycolate, and oxalate) and deposition of calcium oxalate monohydrate crystals inside the lumen of kidney tubules^36^. In the present study, AKI was evident by significantly elevated serum creatinine and blood urea levels observed after EG administration. BPE significantly reduces blood urea and serum creatinine levels and prevents EG-induced AKI. Proteinuria reflects proximal tubular dysfunction, and it is common in AKI. Interestingly, BPE significantly decreased protein excretion, and thus it might have the potential to prevent the nidus formation for crystal nucleation. This finding is similar to our previous finding that showed propolis's ability to decrease protein excretion caused by EG administration^3^.

 EG increased serum levels of liver enzymes (ALT, AST, GGT, and ALP) normalized by ingestion of BPE or cystone. The effect was most likely due to anti-inflammatory and antioxidant properties^37^.

The maximum BPE effect that mitigates the risk factor for stone formation and crystallization and prevention of AKI and liver injury was noted at a dose of 500 mg/kg b.wt/ day compared with 100 and 250 mg/kg b.wt./day. The mechanism underlying this effect is most likely mediated by antioxidant compounds present in BPE and their antioxidant activity. The presented data showed that BPE possesses potent antioxidant activity against DPPH, ABTS, and ferric reducing power. Furthermore, BPE contains a high quantity of phenols and flavonoids. The result is similar to previously published papers^38,39^. Calcium oxalate stimulates the production of reactive oxygen species and inflammation, and antioxidants' administration protects against nephrolithiasis^40^. It was found that EG causes oxidative stress that was mitigated by the antioxidant catechin^41^.

Studies showed that flavonoids possess diuretic effects, and phenols and flavonoids alleviate AKI, renal fibrosis, and inflammation^30,31,42^. These findings might partly explain the BPE protective effect against EG-induced AKI, proteinuria, and crystalluria. Further studies are currently in progress in our laboratory to explore the mechanism of action in-depth.

In clinical practice, the use of acute kidney injury term due to various reasons (AKI) can be extended to 3 months before using chronic kidney disease. In rodent models, there is no such definition.

Using a high dose of EG causes AKI (rapid increase in serum creatinine and BUN), which might take three weeks in humans to resolve with treatment. Most of the studies conducted in rodents used daily doses of 0.75–1% EG dissolved in the water and allow the animals to drink it for 3–4 weeks^43–46^. At the end of the period, laboratory results showed elevated serum creatinine and BUN which indicates kidney failure. Histopathologic studies showed acute changes in kidney tissues such as tubular dilation, glomerular RBC deposition and damage, interstitial inflammation, and oxalate crystal deposition. There is no definitive sign of chronic kidney damage such as glomerular loss or atrophy, severe tubular destruction, and extensive fibrosis. Therefore, it is still appropriate to use AKI during the period of study.

Inflammatory and oxidative markers are important to help understand the mechanism of action. In clinical practice, the markers used to identify kidney injury and failure are serum creatinine, BUN, urine protein, and histopathology. The current study measures various blood and urine biochemical markers and found that BPE prevents and ameliorates the elevation of the markers and histopathologic changes. This gives clear clue that the improvements are due to the use of BPE when compared to the control and groups not treated by BPE. In future studies, we will include inflammatory and oxidative markers. Furthermore, pharmacodynamic studies should be conducted to identify the potency of the interventions. Therefore, further studies are required to determine the potency of BPE.

In conclusion, BPE possesses significant nephroprotective, anti-urolithiasis, and hepatoprotective activities, evidenced by its ability to decrease urine calcium, phosphate, protein, uric acid, and crystals and increase urine volume and urine magnesium. Interestingly, it prevented AKI and acute liver injury by decreasing blood urea, creatinine, and liver enzymes and increasing urine creatinine and urea and creatinine clearance. Also, it ameliorated EG-induced histopathological changes and crystal deposition in the kidney tissue. The mechanism of action is unknown, but its antioxidant activity and its content of flavonoids and flavones might play a role in its activity. Further studies are required to elicit the mechanism of the action and to test BPE in the other kidney diseases that cause proteinuria such as diabetes. These studies will pave the way for clinical studies.

## Methods

### Collection and Extraction of fresh bee pollen

Fresh bee pollen was obtained from beehives in Sidi kacem, west of Morocco, and was directly stored at − 20 °C. The sample was then extracted by maceration at ambient temperature for one week in ethanol 70% under agitation, then filtered through a Whatman filter paper^47^. The alcohol was removed by vacuum rotary evaporation at 60 °C, then dried at the same temperature. The extract obtained was stored at − 20 °C until analysis. Distilled water was added to get three concentrations (100, 250, and 500 mg/kg b.wt.) given to the animals daily by gavage.

### Experimental animals

Adult male Wistar rats (200–220 g) were obtained from the Animal Housing Breeding Center, Department of Biology, Faculty of Sciences, Fes, Morocco, and used for the experiments. Animals were housed under standard environmental conditions (25** ± **1 °C and 12 h/ 12 h light/dark cycle) and were maintained with free access to water and laboratory rat chow. Ethical approval was obtained from Sidi Mohamed Ben Abdallah University in Fez, the Animal Facility, and the Laboratory of Physiology-Pharmacology & Environmental Health, the Faculty of Science Dhar Mahraz, Fez (01DEC2016). The experiments were conducted following the internationally accepted principles for the care and use of laboratory animals. All efforts were made to minimize animal suffering and the number of animals used. The study was conducted in compliance with the ARRIVE guidelines (http://www.nc3rs.org.uk/page.asp?id=1357).

### Experimental groups

Sixty animals were randomly divided into ten groups as Group I-10: containing six animals each.

Group 1: served as a vehicle untreated control and maintained on regular rat food and drinking water ad libitum.

Group 2: received cystone (500 mg/kg b. wt.).

Group 3: received BPE (100 mg/kg b. wt.).

Group 4: received BPE (250 mg/kg b. wt.).

Group 5: received BPE (500 mg/kg b. wt.).

Group 6: received 0.75% v/v EG in drinking water.

Groups 7–9: received 0.75% v/v EG + BPE at a dose of 100, 250 and 500 mg/kg b.wt. per day respectively.

Group 10: received 0.75% v/v EG + cystone (500 mg/kg b.wt. per day).

BPE and cystone were administrated daily by gavage for 30 days. The dose of cystone was 500 mg/kg b.wt., according to other studies^48,49^. The duration of administration of the interventions used in other studies was 30 days^48–50^. EG (0.75%) in drinking water was used^47,50^. The animals drank the water containing EG, and this was monitored as each animal was housed in a metabolic cage with free access to food and drinking water with EG in a graduated bottle. The doses of PPE were chosen according to other studies^51–53^.

### Collection and analysis of urine samples

Each animal was kept in an individual metabolic cage, and the urine was collected on days 0, 15, and 30 of the study period. The animals had free access to food and drinking water during the urine collection period. The urine was analyzed for volume, pH, calcium, inorganic phosphorus, magnesium, electrolytes, urea, creatinine, and total proteins.

### Urinary crystal study

Urine was collected from all groups after 30 days of the experiment, and microscopic examination was performed to identify urinary crystals.

### Blood analysis

On day 30 of the experiment, all the rats were subjected to anesthesia (diethyl ether), and the blood was collected from the retro-orbital sinus puncture. The serum was separated by centrifugation at 10,000 g for 10 min, and analysis of creatinine, urea, sodium, potassium, magnesium, calcium, phosphate and total proteins were performed. Hepatic function was evaluated by measuring serum alkaline phosphatase (ALP), alanine aminotransferase (ALT), Gamma glutamyl-transferase (GGT), and aspartate aminotransferase (AST). All the analyses were performed by Architect c8000 analyzer using two methods potentiometry and spectrophotometry.

### Kidney histopathology

The animals were sacrificed under anesthesia, and the kidney was removed, cleaned off extraneous tissue, and rinsed in ice-cold physiological saline. The kidney was fixed in 10% neutral buffered formalin, processed in a series of graded alcohol and xylene, embedded in paraffin wax, sectioned at 5 µm, and stained with Hematoxylin and Eosin for histopathological examination. The slides were examined under a light microscope to study the architecture of the kidney and calcium oxalate deposits. The kidneys of each rat were studied for the histopathological process. Many slides were performed, and the clearest ones were studied and pictured. The images were processed with GIMP software to increase the clearness.

### Determination of total phenolic content

Total phenol in the bee pollen was determined by the Foline Ciocalteu colorimetric method, according to Ahn et al.^54^. Fifty µL of BPE solution was mixed with 250 µL of the Folin–Ciocalteu reagent (0.2 N) and 200 µL of (75 g/L) Na2CO3, and the absorbance was measured at 760 nm after 2 h of incubation at room temperature. The experiment was done in triplicates, and the results were expressed as mean ± SD mg equivalent of gallic acid/gram bee pollen.

### Total flavonoid content

The flavonoid content was quantified according to the method described by Miguel et al.^55^. Briefly, 200 µl 2% AlCl3 was added to 50 µl of BPE or standard. After 1 h at room temperature, the absorbance was measured at 420 nm. Quercetin was used as standard, and flavonoid content was expressed as mg quercetin equivalents per gram BPE (mg QE/g). The tests were performed in triplicate, and the results were expressed as mean ± SD.

### Total antioxidant capacity

The total antioxidant capacity in the BPE solution was determined according to the ammonium molybdate colorimetric method of Prieto et al.^56^ Briefly, 1 ml of reagent solution (0.6 M sulfuric acid, 28 mM sodium phosphate, and four mM ammonium molybdate) was added to 50 µl of BPE. The mixture was capped and incubated in a thermal block at 95 °C for 1 h and 30 min. The absorbance of the reaction mixture was measured at 700 nm against a blank. Ascorbic acid was used as the standard calibration, and the results were expressed as milligrams of Ascorbic acid equivalent per gram of BPE.

### Free radical scavenging activity on DPPH

The radical scavenging activity of BPE solution against2,2-diphenyl-1-picrylhydrazyl (DPPH) free radical was measured^57^. Fifty µL of BPE solution was added to 825 µL of ethanolic solution of DPPH. Absorbance measurements were read at 517 nm after 20 min of incubation at room temperature; (A1) Absorption of a blank sample containing water instead of BPE, and (A0) DPPH solution acted as the negative control. The percentage of inhibition was calculated using the formula: [(A0 − A1/A0) *100], and IC_50_ was determined. The tests were repeated in triplicate, and the results were given as the mean ± SD.

### Reducing power (FRAP)

The reducing power was determined according to the method described by Moreira et al.^58^ The BPE solution (50 µL) was mixed with 200 µL of 0.2 M sodium phosphate buffer (pH 6.6) and 200 µL of 1% potassium ferricyanide. The mixture was incubated at 50 °C for 20 min, and 200 µL of 10% trichloroacetic acid, 200 µL of distilled water, and 120 µL of 0.1% of ferric chloride were added. The mixture absorbance was measured at 700 nm. Extract concentration providing 0.5 of absorbance (EC_50_) was calculated from the absorbance graph against extract concentration in the solution. Ascorbic acid was used as a positive control. The tests were repeated in triplicate, and the results were expressed as mean ± SD.

### Scavenging activity of ABTS radical cation

The ABTS radical cation (ABTS +) scavenging activity was measured according to the method described by Miguel et al.^59^. Briefly, the ABTS + radical was generated by the reaction of (7 mM) ABTS aqueous solution with K2S2O8 (2.45 mM) in the dark for 16 h and adjusting the absorbance at 734 nm to 0.7 at room temperature. BPE solution (50 µL) was added to (825 µL) ABTS + solution, and the absorbance was measured at 734 nm 5 min after the initial mixing, using water as the blank. Several concentrations were made, and the percentage inhibition [(A0 − A1/A0)*100] was plotted against phenol content, and IC_50_ was determined (concentration of BPE able to scavenger 50% of ABTS + free radical).

### Statistical analysis

The results were expressed as mean ± standard deviation. The statistical significance was assessed using one-way analysis of variance (ANOVA) followed by post hoc Tukey’s Multiple Comparison Test using Graph Pad Prism 5 software.
